# Drosophila Xpd Regulates Cdk7 Localization, Mitotic Kinase Activity, Spindle Dynamics, and Chromosome Segregation

**DOI:** 10.1371/journal.pgen.1000876

**Published:** 2010-03-12

**Authors:** Xiaoming Li, Olivier Urwyler, Beat Suter

**Affiliations:** Institute of Cell Biology, University of Bern, Bern, Switzerland; Stanford University School of Medicine, United States of America

## Abstract

The trimeric CAK complex functions in cell cycle control by phosphorylating and activating Cdks while TFIIH-linked CAK functions in transcription. CAK also associates into a tetramer with Xpd, and our analysis of young Drosophila embryos that do not require transcription now suggests a cell cycle function for this interaction. *xpd* is essential for the coordination and rapid progression of the mitotic divisions during the late nuclear division cycles. Lack of Xpd also causes defects in the dynamics of the mitotic spindle and chromosomal instability as seen in the failure to segregate chromosomes properly during ana- and telophase. These defects appear to be also nucleotide excision repair (NER)–independent. In the absence of Xpd, misrouted spindle microtubules attach to chromosomes of neighboring mitotic figures, removing them from their normal location and causing multipolar spindles and aneuploidy. Lack of Xpd also causes changes in the dynamics of subcellular and temporal distribution of the CAK component Cdk7 and local mitotic kinase activity. *xpd* thus functions normally to re-localize Cdk7(CAK) to different subcellular compartments, apparently removing it from its cell cycle substrate, the mitotic Cdk. This work proves that the multitask protein Xpd also plays an essential role in cell cycle regulation that appears to be independent of transcription or NER. Xpd dynamically localizes Cdk7/CAK to and away from subcellular substrates, thereby controlling local mitotic kinase activity. Possibly through this activity, *xpd* controls spindle dynamics and chromosome segregation in our model system. This novel role of *xpd* should also lead to new insights into the understanding of the neurological and cancer aspects of the human *XPD* disease phenotypes.

## Author Summary

Mutations in human *xpd* cause three different syndromes—XP (xeroderma pigmentosum), TTD (trichothiodystrophy), and CS (Cockayne syndrome)—and various different phenotypes, such as sun-induced hyperpigmentation of the skin, cutaneous abnormalities, neuronal degeneration, and developmental retardation. In addition, while some mutations cause a highly elevated cancer risk, others do not. The multitask protein Xpd functions in transcription, nucleotide excision repair (NER), and in cell cycle regulation. In a situation where transcription is not required and NER not induced, we specifically analyzed the cell cycle function of Xpd in Drosophila. In this situation Xpd locally controls the dynamic localization of Cdk7, the catalytic subunit of the Cdk activating kinase (CAK) to and away from its cellular targets, thereby regulating mitotic kinase activity and mitotic exit. Xpd also controls spindle dynamics to prevent formation of multipolar and promiscuous spindles and aneuploidy. Through multitask proteins like Xpd and Cdk7 cells regulate different cellular pathways in a coordinated fashion. In addition to the basic research relevance, the newly gained knowledge about the cell cycle function of Xpd and its control of spindle dynamics is also relevant for human *xpd* patients because it shows a possible pathway that could lead to highly increased cancer risk and neurological defects.

## Introduction

Metazoan Cdk7 regulates cell cycle progression as the major Cdk-activating kinase (CAK) that is active in vivo [1]–[3]. As a subunit of TFIIH (transcription factor IIH), the CAK complex consisting of Cdk7 (Cyclin dependent kinase 7), CycH and Mat1 also functions in transcription by phosphorylating the CTD (Carboxy-Terminal Domain) of the largest subunit of RNA polymerase II [4],[5]. It appears that this dual role of Cdk7 involves the phosphorylation of different substrates by different kinase complexes. In vitro phosphorylation studies using mammalian kinase complexes showed that free CAK activates cell cycle Cdk targets, whereas TFIIH-associated CAK acts in transcription by phosphorylating RNA polymerase II [6]–[8]. These two different types of substrates share no obvious resemblance, but Cdk7 has evolved two distinct mechanisms to recognize these structurally dissimilar substrates [6].

Xpd (xeroderma pigmentosum group d) is one of two helicases in the TFIIH complex. Its contribution to the transcription function of TFIIH depends, however, on its structural properties and does not require its enzymatic function (reviewed in [9]). Structural, biochemical and genetic data indicate that Xpd links the CAK complex to the core TFIIH complex, and Xpd can be found associated with either the core TFIIH or the CAK complex [7], [10]–[15]. The organizing role of Xpd makes it an excellent candidate for a regulator of the activity of the trimeric CAK complex and its dual function. Indeed, a genetic screen in Drosophila led to the identification of a novel role for Xpd in cell cycle regulation [10]. Xpd negatively regulates the cell cycle function of Cdk7, the CAK activity. Excess Xpd titrates CAK activity, resulting in reduced Cdk T-loop phosphorylation, mitotic defects and lethality, while reduction of Xpd results in elevated CAK activity and increased cell growth and proliferation. Moreover, in blastoderm embryos and S2 cells Xpd seems to be redistributed and possibly down-regulated in the early part of mitosis when Cdk1, a cell cycle target of Cdk7, is maximally active. Down-regulation of Xpd thus seems to contribute to the up-regulation of mitotic CAK activity.

The initial development of the Drosophila embryo is characterized by 13 nuclear division cycles that are rapid, consecutive, nearly synchronous and consist of only S and M phases. Fluctuation of Cdk1 activity in these division cycles is different from that of the typical somatic cell cycle (reviewed in [16]). During the first seven cycles, overall Cdk1 activity in the embryo seems to be stable and not regulated by T-loop and N-terminal phosphorylation. Localized Cdk1/CycB activity is, however, reduced during anaphase, presumably by local degradation of CycB [17]. During cycle 8–13, overall Cdk1 activity starts to fluctuate as gradually more maternal CycB is being degraded during each cycle. In addition, inhibitory Tyr15 phosphorylation by Wee1 also regulates mitotic entry during this stage [18] and the T-loop phosphorylation state of Cdk1 starts to fluctuate in early Drosophila embryos after nuclear cycle 8 [19], suggesting a role of Cdk7/CAK in this stage of development.

Zygotic transcription is not required for proper nuclear division during early embryonic development of Drosophila [20]. This stage would therefore be ideal to further clarify the cell cycle function of Xpd in a situation where we can tell a cell cycle function apart from the transcriptional function. To be able to study the cell cycle function of *xpd* in vivo, we therefore set out to produce embryos that lack Xpd during the early stages of development. Drosophila nuclear division cycles depend on maternal products being loaded into the oocyte before egg deposition. A genetic analysis of the role of *xpd* in regulating these embryonic cell cycles therefore involves the production of embryos that do not receive functional maternal Xpd. In this work we present the isolation of an *xpd* mutant and the production of Xpd-deprived embryos that we use to analyze the cell cycle function of *xpd* in preblastoderm and syncytial blastoderm embryos. Mutant embryos show abnormal subcellular distribution of Cdk7, abnormal anaphase inactivation of Cdk1, a mitotic substrate of Cdk7, and they loose the division synchrony and show nuclear division defects including faulty segregation of chromosomal material. The results show that Xpd functions in nuclear division and they suggest that it does this via controlling the subcellular localization of CAK activity and spindle dynamics. In this process, the re-localization of CAK away from the chromosomes and Cdk1 seems to be essential for timely coordinated down-regulation of Cdk1 activity to allow synchronous exit from mitosis.

## Results

### Drosophila *xpd* is an essential gene, and Xpd::V5 provides *xpd* activity

**Figure 1:**
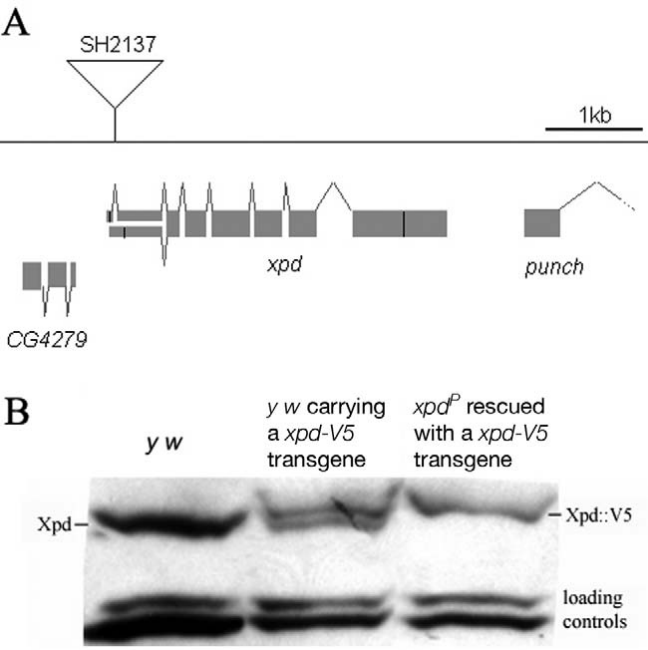
*xpd* gene and alleles. (A) Map of the *xpd* genomic region. Known transcripts and the P-element insertion (triangle) in *xpd* are shown (figure based on the information provided by www.flybase.org). Boxes depict exons, connecting lines introns of the different transcripts. The alternative exons at the 5′ end of *xpd* are visualized by split boxes, with the two different translational starts shown as black vertical bars. The position of the translational stop codon in the last exon is also indicated by a black bar. (B) *xpd-V5* rescues the *xpd^P^* mutant phenotype. Total fly extracts were analyzed by Western blotting with anti-Drosophila Xpd antibodies. *xpd-V5* transgenic lines express similar levels of exogenous and endogenous Xpd. Unidentified cross reacting bands serve as loading controls. In *xpd^P^* flies rescued with *xpd-V5*, only Xpd::V5 is detected.

The homozygous lethal line *l(2)SH2137* contains a P-element insertion 7 nucleotides upstream of the 3′-splice junction of the first intron of *xpd* and was therefore named *xpd^P^* (Figure 1A). *xpd^P^* is also lethal over the chromosomal deficiency *Df(2R)K11*, which uncovers the *xpd* locus, and a wild-type genomic *xpd* transgene restores viability and fertility to hemi- and homozygous *xpd^P^* flies (*w P[w^+^ xpd^+^]/w; xpd^P^*/*Df(2R)K11* and *w P[w^+^ xpd^+^]/w; xpd^P^*/*xpd^P^*; Supporting Table S1). *xpd* is therefore an essential gene in Drosophila and rescue of the lethality caused by the *xpd^P^* mutant serves as an excellent test for the in vivo functionality of different modified *xpd* transgenes, including tagged versions of *xpd*. Two independent integrations of a fly genomic *xpd-V5* transgene can restore viability to the hemizygous *xpd^P^* mutant (*w P[w^+^ xpd-V5]/w; xpd^P^*/*Df(2R)K11*; Supporting Table S1). However, the V5 tag seems to somewhat reduce *xpd* activity and we only observe about 1/4 (between 1/3 and 1/5) of the expected flies of this rescued class. In contrast to the V5 tag, the fusion of a fluorescent protein, either green (GFP) or yellow (YFP) fluorescent protein, to the C-terminus of Xpd abolished its function, even though the C-term of Xpd seems to be on the surface of the TFIIH complex [15]. None of three independently derived and expressed *xpd-GFP* or two *xpd-YFP* transgenic lines with different insertion sites rescued the lethality of the hemizygous *xpd^P^* mutant (data not shown).

Flies carrying a V5-tagged *xpd* transgene in the hemizygous mutant background (*w P[w^+^ xpd-V5]/w P[w^+^ xpd-V5] (or w); xpd^P^*/*Df(2R)K11*) were used to determine whether Xpd protein is still synthesized from the *xpd^P^* allele. The Xpd fusion protein can be distinguished from the wild-type gene product by its slower migration through polyacrylamide gels (Figure 1B). On Western blots, no Xpd protein with wild-type electrophoretic mobility could be detected in extracts of whole flies or ovaries of these rescued mutant flies. Because the epitope used to produce the antibody is encoded by sequences downstream of the P-element insertion site, we conclude that the P-element insertion in the first intron of *xpd* disrupts the expression of *xpd*. Taking this result together with the genetic characterization of *xpd^P^*, this allele behaves as a null mutant.

### Making embryos that specifically lack Xpd

**Figure 2:**
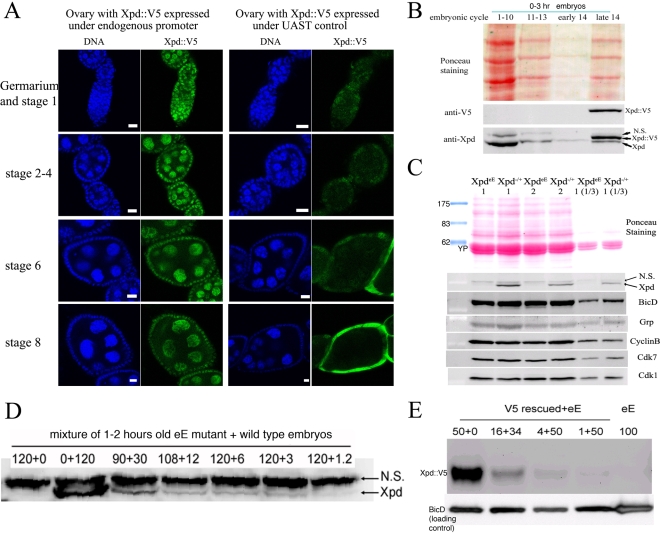
Production of Xpd-deprived embryos. (A) Expression of Xpd::V5 from the endogenous *xpd* promoter is readily seen in the germ line where the signal becomes localized to the large nurse cell nuclei. In contrast, tub-Gal4 - UAST driven Xpd::V5 expression is much lower in the germ line, but the Xpd::V5 signal is readily seen in the cytoplasm of stage 8 follicle cells (all pictures were taken with the same laser power and scanner settings). Ovaries expressing Xpd::V5 under the endogenous promoter are from *w; xpd^P^; P[w^+^xpd-V5]* flies, and ovaries expressing Xpd::V5 under UAST control are from *w; xpd^P^; tub-Gal4/UAST-xpd-V5* flies. Anti-V5 antibody was used for staining and scale bars represent 10 µm. (B) Zygotic expression of Xpd from the transgene is only detectable starting in late cycle 14. Embryos laid from *w; xpd^P^/CyO; tub-Gal4/UAST-xpd-V5* mothers expressing both endogenous Xpd and transgenic Xpd::V5 under UAST control were stained with Hoechst and staged. Extracts of embryos at different stages were separated by SDS-Page and probed with anti-V5 and anti-Xpd. “N.S.” stands for non-specific cross reactive band. (C) No Xpd can be detected in *xpd^eE^* embryos, while other maternal proteins were loaded normally into *xpd^eE^* embryos. Extracts of 35 0–30 min *xpd^eE^* embryos or control embryos from *w; xpd^P^/CyO; tub-Gal4/UAST-xpd-V5* mothers were loaded on each lane of a Western blot and probed with different antibodies. 1 and 2 indicate two samples collected at different times for each type of embryo. “1/3” means that one third of amount of the extract was loaded. YP is yolk protein and the masses of the markers are indicated in KDa. (D) Quantification of Xpd levels (from *xpd^P^*) in *xpd^eE^* embryos mixed with wild type embryos at different ratios. Numbers above lanes indicate ratios of *xpd^eE^* embryos mixed with control embryos with a functional *xpd^+^*. Even at ratios of 100∶1 (120+1.2), a faint Xpd band is still visible. Because no band is seen in this position in *xpd^eE^* embryos, Xpd levels in this mutant must be below 1% of normal levels. (E) Quantification of Xpd::V5 levels in *xpd^eE^*. Anti-V5 antibody staining of a Western blot showed that no Xpd::V5 could be detected from 100 *xpd^eE^* embryos, while it could be detected from even one single *xpd^−^* embryo rescued with a *xpd-V5* transgene expressed under its endogenous *xpd* promoter after the sample was diluted 50 times (1+50).

Direct testing of potential cell cycle functions of *xpd* in the absence of a transcriptional requirement should be possible if we can make young preblastoderm embryos that lack Xpd. During the rapid nuclear divisions of the preblastoderm stage, embryonic transcription is not required for development and survival. Towards producing such embryos we constructed mothers that express *xpd* in the soma only. For this we first made transgenic flies that express Xpd under UAST control [21]. UAS (“upstream activating sequence”) is a transcriptional enhancer element from yeast that is normally inactive in *Drosophila*. It can be activated in a controlled manner by introducing into the fly the transcription factor Gal4 under control of a promoter of choice. The UAST system contains a *hsp70* basal promoter to direct transcription initiation downstream of the UAS enhancer. This basal promoter is not active on its own, but needs to be combined with enhancer elements. In this combination the system allows good expression in the somatic tissue, but does not support expression in the germ line during the vitellarial stages of oogenesis, probably due to poor activation of the *hsp70* basal promoter in this tissue [22]. To find out whether it was possible to produce embryos that lack Xpd, we studied the expression of *UAST-xpd-V5* in the *xpd^P/P^* mutant background. Consistent with the expectation, we found that V5-tagged Xpd expressed under UAST control with a tubulin-Gal4 driver accumulated in the female germ line to a much lower degree than Xpd-V5 expressed under its endogenous promoter (*w; xpd^P^; tub-Gal4/P[w^+^ UAST-xpd-V5]* and *w; xpd^P^; P[w^+^xpd-V5]* ovaries, Figure 2A). In the somatic follicle cells, however, *UAST-xpd-V5* is highly expressed from stage 8 onward. To monitor maternal deposition and zygotic accumulation of Xpd in the embryo, we analyzed embryos from mothers containing the transgenic *UAST-xpd-V5* and one copy of endogenous *xpd^+^* (required for development, see below; *w; xpd^P^/CyO; tub-Gal4/UAST-xpd-V5*). Early embryonic stages from nuclear cycle 1–13 do not show any Xpd-V5 expression and zygotic expression becomes detectable just prior to the start of mitosis in nuclear cycle 14 (Figure 2B).

Next, we tested whether expression of the *UAST-xpd-V5* transgene driven by *tub-Gal4* could rescue the lethal phenotype of *xpd^P^* flies. Indeed, *xpd^P^; tub-Gal4/UAST-xpd-V5* flies develop to the imago stage. Adult rescued flies do not display any abnormalities and rescued males are fertile. Rescued females show normal egg production, suggesting that oogenesis proceeds normally (data not shown). The resulting embryos produced by homozygous *xpd^P^* females with a *tub-GAL4* driven *UAST-xpd-V5* construct (*xpd^P^; tub-Gal4/UAST-xpd-V5*) will be called *xpd^eE^* embryos (for early embryos). While these embryos initiate development normally, no larvae emerged from them, and neither transgenic Xpd::V5 nor endogenous Xpd protein could be detected on Western blots from laid embryos (Figure 2C). As determined by a dilution series, they contain less than 1% of the normal Xpd or Xpd-V5 levels (Figure 2D and 2E). To find out whether the reduced levels of Xpd in the germ line lead to accumulation of reduced levels of other cell cycle regulators active in the embryo, we also measured levels of Cdk1, Cdk7, CycB, and Grp (Grapes) in *xpd^eE^* embryos (Figure 2C). Surprisingly, despite the massive transcription requirement in the nurse cells and despite the drastically reduced Xpd levels in the female germ line, normal levels of these cell cycle regulators (and other proteins) were loaded into the eggs and young embryos. Either residual Xpd levels are still sufficient for normal transcription or there is a less strict requirement for *xpd* for transcription in the older germ line. Because similar results were obtained with *p52* (encoding a core TFIIH component) mutants and mutant clones that are capable of supporting transcription and development for several days (Holenstein and Suter, unpublished), [23],[24], this suggests that the general transcription factor TFIIH may not be as “general” a transcription factor as previously thought.

### Division defects, chromosomal instability, and uncoordinated division cycles in *xpd^eE^*

**Figure 3:**
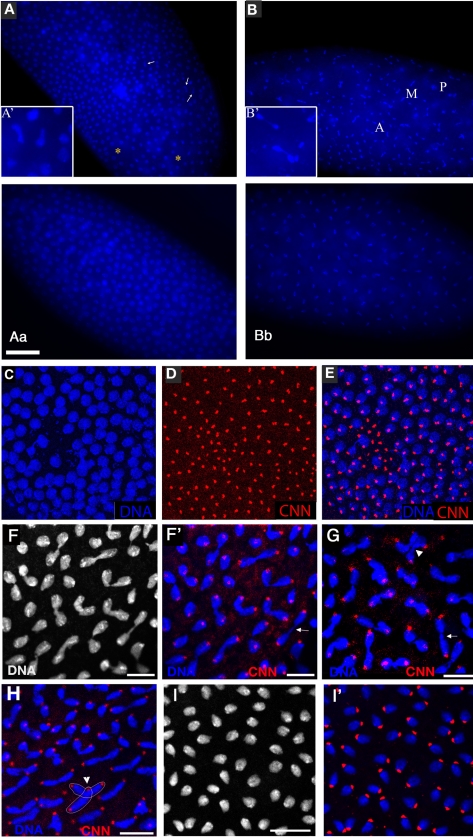
*xpd^eE^* embryos show nuclear division defects, loss of division synchrony, and chromosomal instability. (A) Arrows point to undivided nuclei in a cycle 12 *xpd^eE^* embryo. “*” marks regions without nuclei. Staining was with Hoechst and polyploid yolk nuclei in the center of the embryo are also visible in this epifluorescence micrograph. (Aa) Wild-type interphase 12 control. Bar is 50 µm. (B) This cycle 11 *xpd^eE^* embryo shows unsynchronized nuclear divisions. P: prophase; M: metaphase; A: Anaphase. Nuclear fusion (A′) and chromatin bridges (B′) are frequently seen in the mutant embryos. (Bb) Wild-type metaphase 11 control showing the tight cell cycle synchronization of the nuclei on the surface. (C–E) Part of an *xpd^eE^* embryo stained with Hoechst (blue, DNA) and anti-CNN antibody (red) to visualize centrosomes. The region lacking nuclei shows free centrosomes. (F–H) Incompletely segregated chromosomes are partially or fully decondensed (note the differences in condensation between the centromeric heterochromatin and the euchromatin that is decondensed). DNA is stained with Hoechst (white and blue). Note the banana shaped mitotic figures. (F′–H) Additional CNN staining marking the centrosomes is shown in the red channel. Arrows point to chromosomal material left back at the central spindle. Arrowheads point to dividing chromosomes from neighboring mitotic figures that are closely associated (G) and to a situation where two neighboring mitotic figures cross one another (also outlined in H). (I) Wild-type (*xpd^−/+^*) control embryos of the same stage stained with Hoechst. (I′) Same control embryos as in (I) also stained for CNN in red. Scale bars represent 10 µm.

During the nuclear division cycles *xpd^eE^* embryos show high rates of nuclear division defects (Figure 3). Concomitant with these defects, the synchronization of late syncytial division cycles is also lost in many embryos. Under our conditions, only 11.6% (n = 354) of 1–2 hour old *y w* wild type embryos show some sort of division defects or asynchrony at the time of fixation. Furthermore, these defects are always relatively minor and usually restricted to a small region in the embryo. In contrast, 38.7% (n = 382) of total 1–2 hour old *xpd^eE^* embryos show nuclear division defects. This difference becomes even more impressive when one considers that we see the defects primarily during the mitotic phase. Defects can be restricted to only small regions of the embryo (Figure 3A) or can affect large parts of the embryo (“severe defects”, Figure 3B). The severe defects are only seen in *xpd^eE^* embryos and are fully rescued by a transgenic copy of genomic *xpd* because no single embryo laid by *xpd^P^* mothers rescued by one copy of a genomic *xpd^+^* transgene showed severe defects (n = 108; *xpd^P^*; t*xpd^+^*/*tub-Gal4 or TM3 Ser*). Similarly, no embryo from mothers of the genotype *xpd^P^*/+ (CyO); *UAST-xpd*/*tub-Gal4* showed such defects. In *xpd^eE^* embryos defective nuclei failed to segregate their chromatin properly and displayed nuclear fusions and chromatin bridges (arrows in Figure 3A and 3B; magnified in 3A′, 3B′; compare to respective wild types 3Aa and 3Bb). Chromatin bridges can be seen in interphase (A), in late anaphase or telophase (B); incompletely segregated chromosomes that are fully decondensed are shown in Figure 3C–3E and partially decondensed ones in Figure 3F. Often we also observe bent (banana shaped) dividing chromosomes that fail to form a straight segregation axis and we observe mitotic figures in which sister chromosomes have not completely segregated yet (Figure 3F–3H). Incomplete segregation is even seen in mitotic figures that do not show the characteristic anaphase orientation anymore (with centromeres towards the spindle pole and telomeres lagging behind). This indicates that the spindle forces stopped pulling the chromosomes apart and telophase had been reached. Therefore, these observations show that many nuclei failed to properly segregate their chromosomes by telophase. This staging interpretation is also consistent with the finding that these chromosomes are not stained with the mitotic marker antibody against phospho-Histone H3 (PH3), indicating that mitotic kinase activity has already dropped in these mitotic figures (data not shown). Often this phenotype is combined with chromosomal material remaining left back at the central spindle in late mitosis (arrows in Figure 3F′, 3G) and with spatial organization problems, where dividing chromosomes from neighboring mitotic figures are closely associated (see arrowhead in Figure 3G) or where two neighboring mitotic figures cross one another (arrowhead and outline in Figure 3H), something that usually does not happen in wild type animals (Figure 3I, 3I′).

Chromatin-free regions can also be seen in *xpd^eE^* embryos (“*” in Figure 3A). In these regions we observe free centrosomes (Figure 3C–3E), suggesting that improperly divided nuclei fell into the interior of the embryo during previous cycles and that the centrosomes were left at the cortex. Some of the extra centrosomes can be seen associated with nearby nuclei (Figure 3E). In cases where different division phases become apparent in large parts of the embryo (Figure 3B), the phenotype indicates a general loss of division synchrony and/or a defect in the propagation of the mitotic wave that is typical for the late syncytial stages. As the severe nuclear division defects, the loss of synchrony can be rescued with a transgenic genomic copy of *xpd^+^* crossed into the *xpd^eE^* background.

**Figure 4:**
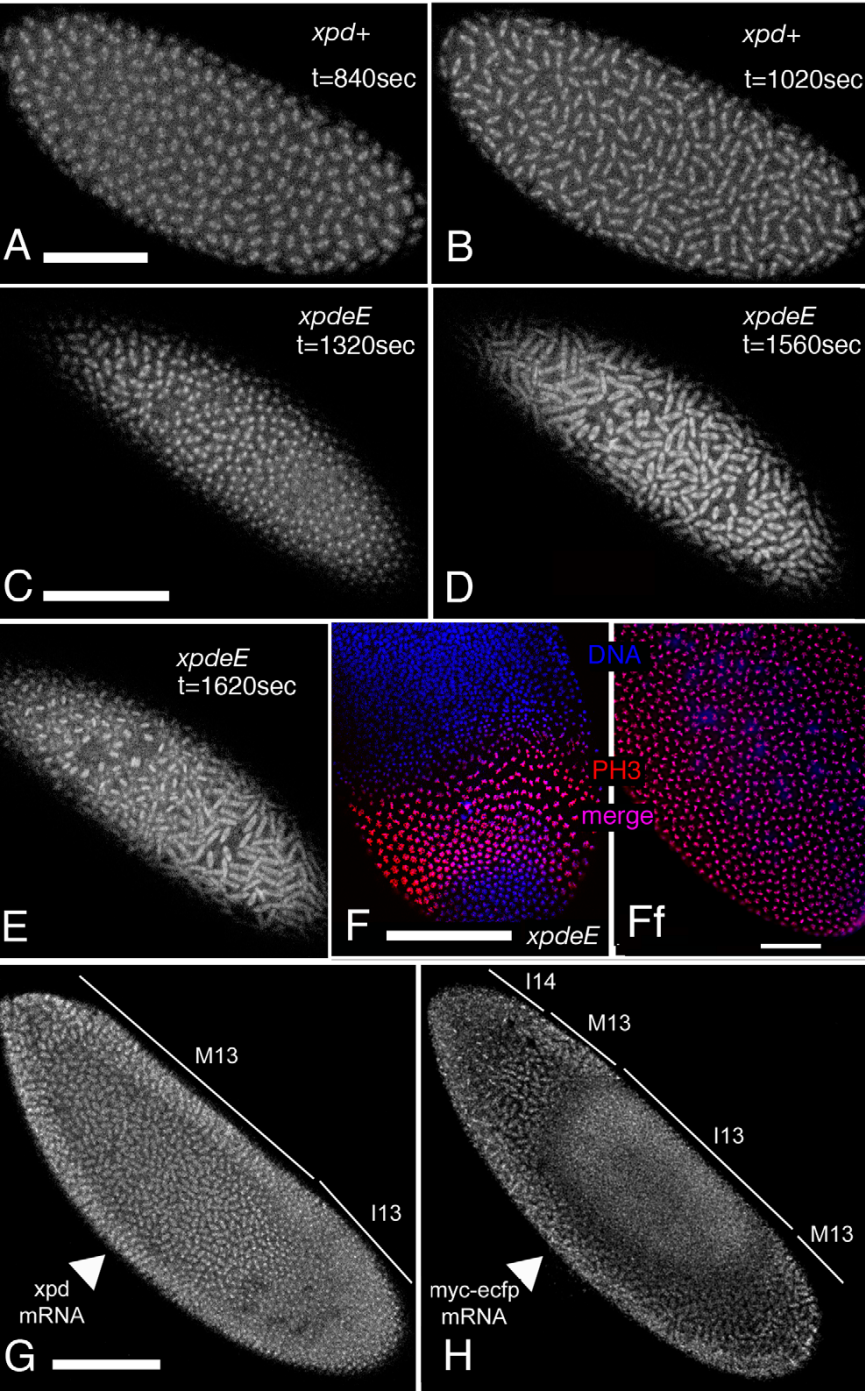
Defects in the nuclear division cycles and the mitotic wave in *xpd^eE^* embryos. (A–E) Projections of four optical sections from the confocal time-lapse movies (Video S1 and Video S2) showing embryos at single time points in nuclear cycle 12. The mitotic spindle is visualized with the gene trap line G147. (t) indicates the time that elapsed since the beginning of the recording. Anterior is to the left. (A,B) Embryos from mothers with a functional *xpd^+^* gene. (C–E) *xpd^eE^* embryos enter metaphase and anaphase first at the anterior pole while the posterior region lags behind. (F) Posterior region of an *xpd^eE^* embryo in interphase of nuclear cycle 13 (bottom) and interphase 14 (top) stained for DNA (blue) and the mitotic marker PH3 (red). The anterior mitotic wave has almost reached the posterior end. (Ff) Wild-type (*xpd^P^/+; UAST-xpd-V5/tub-Gal4*) control. (G,H) Rescue experiment of mitotic wave defect by injection of *xpd^+^* mRNA into the center of an *xpd^eE^* embryo (G). Note the extended mitotic region compared to (F). Injection of the control mRNA *myc-eCFP* into the center of an *xpd^eE^* embryo does not rescue the wave, but further delays it locally (H). Cell cycle stages are indicated: M = mitotic phase, I = interphase, 13 and 14 indicate the nuclear cycle. Arrowheads point to the ventral site of injection. Pictures are projections of four surface sections from a live imaging experiment. Scale bars represent 100 µm, except for (Ff), where it is 50 µm.

To analyze the embryonic mitotic cycle in more detail we took advantage of the third chromosome G147 gene trap line, in which GFP is expressed in frame with a microtubule associated protein and therefore marks the mitotic spindle [25]. Time-lapse analyses of embryos expressing G147 show a loss of mitotic synchrony in the late nuclear division cycles of *xpd^eE^* embryos (Figure 4; compare Video S1 with Video S2). During the later, metasynchronous, nuclear division cycles of wild type embryos, the mitotic waves start from both poles and move rapidly towards the center of the embryo (Figure 4A, 4B). In *xpd^eE^* embryos nuclear cycles 12 and 13 usually take much longer than in the wild type. While we often observe delays of 50–100% at the anterior end of the embryo, the delays at the posterior can even be much longer. The same time-lapse analyses show that the mitotic wave from the anterior pole often proceeds almost or entirely to the posterior end before the posterior mitotic wave is initiated (Figure 4C–4E; compare Video S1 with Video S2). This situation was seen in 10/14 *xpd^eE^* embryos and was rescued by one copy of transgenic genomic *xpd^+^* (detailed genotypes are described in Materials and Methods). Only 1 of 11 rescued embryos showed such a defect and this was even a very mild one. A slowly progressing mitotic wave from the anterior combined with the lack of a posterior wave was also visualized in fixed embryos (Figure 4F, compare to 4Ff). Note also that there are regions in the mutant that lack mitotic spindles (Video S2 and Figure 4C–4E). We conclude that embryos need Xpd also for normal progression through the rapid mitotic cycles and for the coordination and timely initiation of the mitotic wave emanating from the posterior pole. This defect in propagation of the mitotic wave could reflect an indirect requirement for Xpd for maternal transcription of another factor or it could reflect a more direct function of Xpd in cell cycle regulation. If delivery of *xpd* mRNA directly into the *xpd^eE^* embryos was able to rescue the phenotype, this would argue against a maternal transcription defect. We therefore injected *xpd* mRNA and, as control, a mRNA that encodes a myc-tagged *eCFP (myc-*e*CFP)* into the center of *xpd^eE^* embryos and monitored the progression of the mitotic waves. In 4 out of 5 injected embryos, injection of *xpd mRNA* caused the nuclei in the vicinity of the injection site to enter mitosis in a more timely fashion and earlier than the ones further posterior to the injection site, suggesting that the *xpd* mRNA injection rescued the propagation of the mitotic wave locally (Figure 4G). In contrast, control injections did not rescue the mitotic wave defect, but tended to further delay the progression of the mitotic wave around the injection site (n = 6; Figure 4H).

### Xpd safeguards mitotic chromosomes from abduction by neighboring spindles

**Figure 5:**
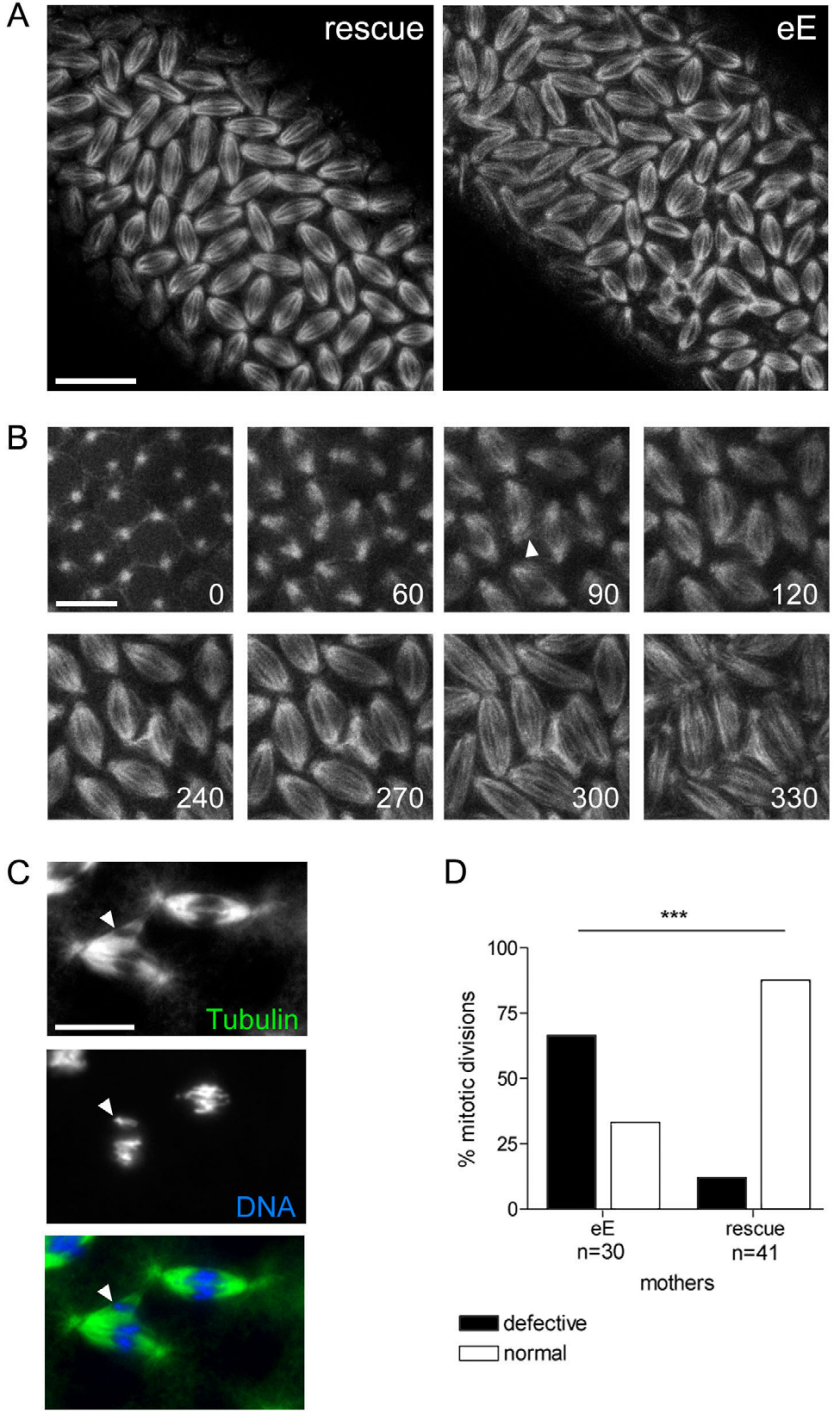
Trespassing microtubules attach to neighboring chromosomes. (A) Mitotic spindles during cycle 13 visualized with G147. Pictures are projections of four optical sections of live imaged control (rescue) and *xpd^eE^* embryos (eE). For the latter, the time point shown is the very first frame that was recorded for this embryo. Scale bar represents 20 µm. (B) Projections of four optical sections from a time series of a different *xpd^eE^* embryo in cycle 13. Time is indicated in seconds from the first frame. Arrowhead indicates microtubule fibers that are faultily targeted towards the neighboring nuclear region as the spindles form. Scale bar represents 10 µm. (C) Neighboring mitotic figures in nuclear cycle 11 stained for DNA and Tubulin. Arrowhead points to chromatin that is separated from the rest of the chromosomes and contacts microtubule fibers emanating from a neighboring spindle. Scale bar represents 10 µm. (D) Mitotic division waves were monitored for 14 *xpd^eE^* embryos and 15 rescue embryos from nuclear cycle 10–13. Embryos were scored as defective if in the imaged area one or more spindle contacts with neighboring spindles were observed; ***, the difference is significant, p<0.0001 (Fisher's exact test).

To learn more about the mechanism of Xpd action during the division cycles, we made high magnification time-lapse movies from embryos expressing the G147 gene trap line to visualize the microtubules during mitosis (Figure 5). As controls we used the *xpd^eE^* embryos that additionally express a transgenic *xpd^+^*. Compared to the rescued control the *xpd^eE^* embryos showed a highly elevated frequency of mitotic spindles that branch out into neighboring mitotic figures (Figure 5A). The still image of the *xpd^eE^* embryo shown in Figure 5 is the first frame that was acquired for this embryo, ruling out photo toxicity effects. GFP labeled fibers can first be seen contacting neighboring spindle regions around prometaphase and stable attachments are being formed that cause one spindle to move with the attached part of the second spindle during ana- and telophase (Figure 5B). To find out whether the fibers that crosslink two spindles are indeed microtubule fibers and to find out what happens to the chromosomes in these situations, we also stained embryos simultaneously for DNA and Tubulin. As shown in Figure 5C, microtubules from the “thief” spindle reach over to the neighboring mitotic figure, where they seem to attach to a chromosome. The position of this chromosome suggests that it was strongly attached to the “thief” spindle and one pole of its normal spindle, and that the “thief” spindle forces were able to pull it sideways out of the metaphase plate. These defects are caused by reduced *xpd* activity because they can be rescued by expressing wild type *xpd^+^* in this genetic background (Figure 5D).

### Xpd controls subcellular Cdk7 localization and local Cdk1 activity

**Figure 6:**
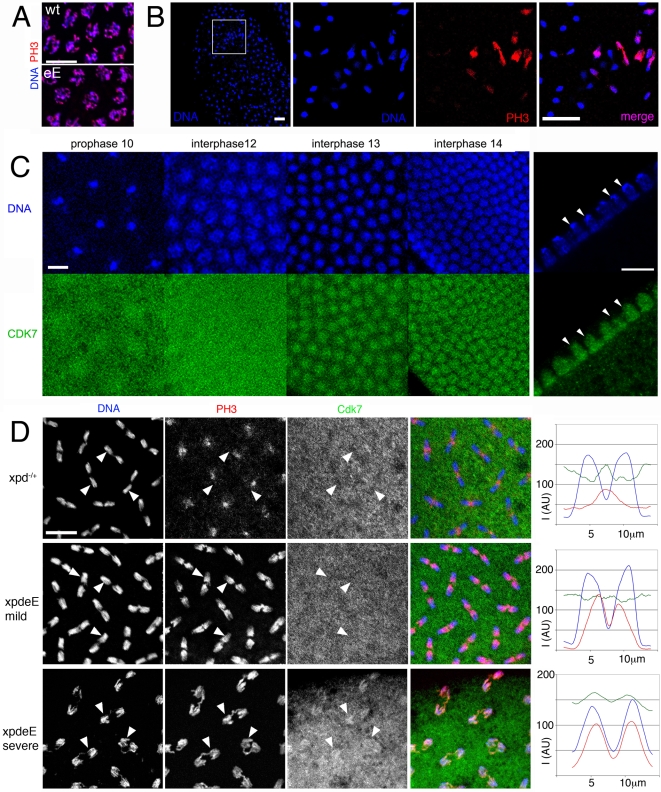
Disruption of dynamic subcellular localization of Cdk7 and of Cdk1 activity in *xpd^eE^* embryos. (A) PH3 staining of wild type (wt: *xpd^−/+^*) and *xpd^eE^* (eE) embryos does not reveal differences in Cdk1 activity during a normally initiated prophase. Scale bar represents 10 µm. (B) Local mitotic defects and loss of cell cycle synchronization in *xpd^eE^* embryos. Some areas lack chromosomal DNA (left panel, outlined area is shown magnified in the adjacent panels), while others show interphase chromosomes (blue, lacking red PH3 staining) side by side with patches of mitotic chromosomes that stain for PH3 (red). The embryo also shows ana- and telophase figures where PH3 staining persists too long. Wild type telophase figures do not stain for PH3 anymore (not shown). Scale bar represents 20 µm. (C) Cdk7 staining of syncytial wild type embryos changes during the later cycles. Cross-section through interphase 14 nuclei (right panels) reveals that Cdk7 is depleted from heterochromatic regions that strongly stain for the DNA dye Hoechst (arrowheads; scale bar 10 µm). (D) Polar loss of PH3 staining (red) from cycle 12 anaphase chromosomes (blue) is seen in embryos with Xpd (heterozygous *xpd^−/+^*were used here), where Cdk7 (green) gets slightly excluded from the chromosomal region (upper panels). Arrowheads allow following individual chromosomal regions in the different channels. In the mild defects seen in *xpd^eE^* embryos PH3 staining is seen on the central half of the anaphase chromosomes and Cdk7 signal is uniformly distributed (middle panels). In the more severe cases (bottom panels) the entire chromosomes are still PH3 positive and this correlates with a chromosomal enrichment of Cdk7 (bottom panels). Signal intensity for the three different channels was measured along the axis of five anaphase figures. The results are plotted in the right panels. I is intensity and AU are arbitrary units. Scale bar represents 10 µm.

Entry into mitosis and progression to metaphase requires activation of Cdk1 and exit from mitosis requires timely inactivation of this kinase. The observed mitotic defects could be caused by reduced Cdk1 activity or by a hyperactive Cdk1. In general, *xpd^eE^* embryos seem to build up Cdk1 activity normally as individual nuclei enter mitosis. Western blotting shows that Cdk1, CycB and Cdk7 are deposited normally in *xpd^eE^* embryos (Figure 2C), and Histone H3 phosphorylation (PH3), a read-out for local Cdk1 activity [17], appears normal in most prophase nuclei of *xpd^eE^* embryos (Figure 6A). It therefore seems that Cdk1 activity is generally properly initiated as nuclei enter mitosis, although we also observe chromosomes that fail to stain for PH3 (Figure 4F and see below). Another cause for prolonged mitosis and chromatin bridges (Video S2, Figure 3, Figure 4) could be a failure to reduce mitotic Cdk activity in *xpd^eE^* embryos. In early Drosophila embryos, proper exit from mitosis requires localized inactivation of Cdk1, which can be visualized by monitoring Histone H3 phosphorylation (PH3) [17]. Consistent with the time-lapse observation, wild type syncytial embryos show synchronized PH3 staining and de-staining (data not shown). However, this is not the case for *xpd^eE^* embryos, where we often see smaller or larger regions with extended PH3 staining (Figure 6B). In this embryo, most nuclei have entered late telophase or early interphase and do not show PH3 staining, while some nuclei are still in ana- or telophase and show strong PH3 staining. We further noticed that in *xpd^eE^* embryos the PH3 signal often persists over the entire chromosomes through anaphase whereas it gets restricted to the telomeric regions during wild type anaphase (see also below).

Because reduced Xpd levels can cause an increase in CAK activity, the cell cycle function of Cdk7 [10], we tested if CAK activity changes in *xpd^eE^* embryos. Possibly because of the developmental heterogeneity in the embryonic samples, we were not able to show a convincing CAK activity increase in extracts of *xpd^eE^* embryos (data not shown). Because overall Cdk7 protein levels also seem to be normal in these mutant embryos (Figure 2C), we next examined the subcellular localization of Cdk7 in the *xpd^eE^* embryos and controls using monoclonal antibodies against Drosophila Cdk7 [2]. For this localization study we focused on the nuclear division cycles 10–14, when defects are seen in embryos lacking Xpd. In wild type embryos the subcellular distribution of Cdk7 is dynamic and it changes depending on the phase of the mitotic cycle as well as between the early and later cycles (Figure 6C). Until cycle 12, Cdk7 signal is distributed in a relatively uniform pattern throughout the cytoplasm of wild type embryos, with some enrichment concentrically around the DNA region that gives the staining pattern an energid-like appearance during the earlier stages (prophase 10). During interphase of cycles 13 and 14, Cdk7 signal progressively accumulates in the nuclei where it is concentrated in the euchromatic region and depleted from the heterochromatin of the cycle 14 nuclei (Figure 6C, right panels). Cdk7 staining around the chromosomes generally gets lost progressively during mitosis (Figure 6D, upper panels). Differences between our results and previously published ones [26] are probably due to the methanol sensitivity of the Cdk7 epitope that we circumvented with our fixation protocol (see Materials and Methods).

In *xpd^eE^* embryos the PH3 signal is often retained on the entire chromosomes during ana- and sometimes even telophase (Figure 6B and 6D), and fails to get lost in a polar manner from the centromeres. These altered staining patterns show that Cdk1 activity is not properly controlled in the absence of Xpd. During interphase and prophase of these cycles, Cdk7 shows a slight increase in subcellular localization to the chromosomal region. In contrast, in wild type anaphases, this region then displays reduced intensity of Cdk7 signal compared to the surrounding cytoplasmic region (arrowheads in Figure 6D). In the *xpd^eE^* mutant, however, the Cdk7 signal is generally more uniform and not or less reduced in the chromosome region. This pattern correlates with retention of PH3 staining over large parts of the chromosomes (middle panels and arrowheads in Figure 6D). To better visualize these differences in distribution and activity we quantified the signal intensity along a 14 µm line through anaphase figures, starting and ending in the cytoplasm (Figure 6D, right panels). Hoechst fluorescence intensity is shown in blue and shows the position of the chromosomes. In the wild type (*xpd^−/+^*) situation, Cdk7 signal (green) is reduced over most of the anaphase chromosomes except for the central region of the anaphase figure, where lagging telomeres also show elevated PH3 staining (red), leading to a central maximum. In contrast, in the mild phenotypes of *xpd^eE^* embryos Cdk7 signal is relatively constant along the axis and the PH3 signal peaks over the telomeric parts towards the central region of the mitotic figures, resolving into two peaks with a low in-between. In severe mutant phenotypes we observe even an elevated chromosomal association of Cdk7 signal during anaphase and this correlates with retention of the PH3 signal over virtually the entire chromosomes (bottom panels of Figure 6D). On the other hand we also see embryos with reduced anaphase Cdk7 staining that correlates with normal loss of PH3 staining, a situation that is indistinguishable from the wild type and may well correspond to embryos (or regions) that proceed normally through the cycles (data not shown). The good correlation suggests a causal relationship between the lack of Xpd, ectopic Cdk7 localization, lack of Cdk1 inactivation and persistence of PH3 on anaphase chromosomes.

A detailed analysis of the *xpd^eE^* phenotype did not reveal any evidence that the delayed and incomplete dephosphorylation of PH3 in *xpd^eE^* embryos is caused by DNA damage or incomplete replication. First, staining for Tubulin and analysis of the G147 GFP trap line revealed normally shaped and focused spindles in the mutants (Figure 5 and Video S2). Second, γ-tubulin signal is normal even at spindle centrosomes that show division defects (see Supporting Figure S1). DNA damage or replication problems would, however, inactivate the centrosomes and interfere with normal spindle formation [27]. Third, like wild type embryos, *xpd^eE^* embryos still arrest at prometaphase upon colchicine treatment (a microtubule depolymerizing drug; data not shown). This and the lack of additional *bubR1^Rev1^* phenotypes (extensive loss of nuclear synchrony in nuclear cycles 4–5, and precocious sister chromatid separation [28],[29]) suggests that spindle check point proteins are also present and the checkpoint is functional in *xpd^eE^* embryos.

### Cyclin B dependence of nuclear division defects

**Figure 7:**
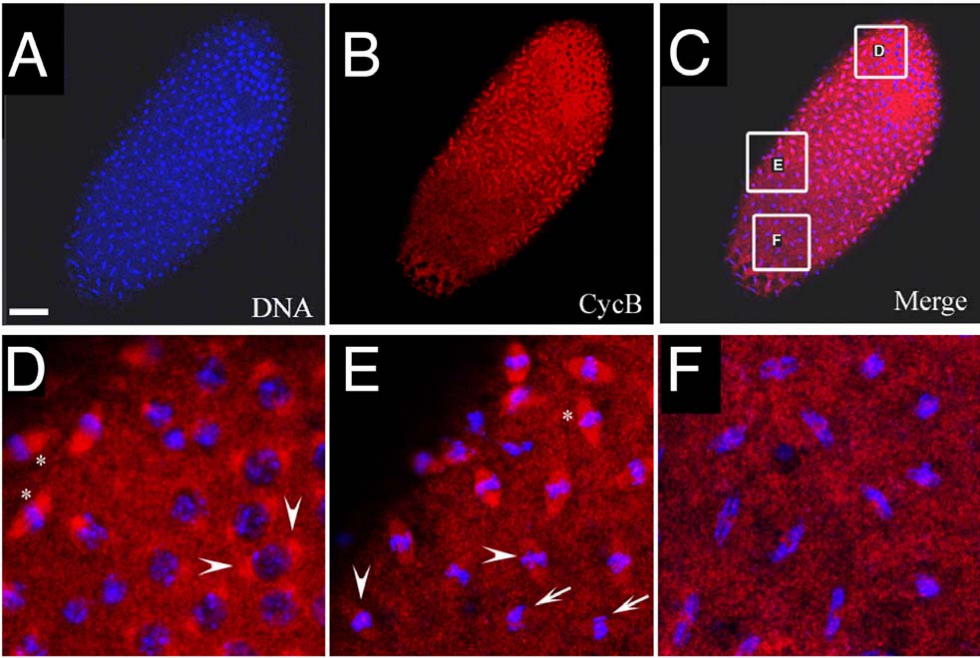
Cyclin B distribution in asynchronous mitoses of *xpd^eE^* embryos. (A–F) The mutant cycle 11 embryo was stained with Hoechst (blue) and anti-CycB (red). Scale bar represents 50 µm. Nuclear divisions (A) and CycB degradation (B) are not synchronized throughout the embryo. However, the latter takes place at the appropriate cell cycle stage. (D–F) show regions from (C) at higher magnification. Arrowheads in (D) show CycB localized to spindle regions of prophase nuclei. “*” in (D,E) show CycB localized to spindle regions and DNA regions of early metaphase nuclei. Arrowheads in (E) show levels of CycB drop in spindle regions as nuclei progress through metaphase. Arrows in (E) show no localization of CycB to spindle regions in late metaphase/early anaphase nuclei. The same is the case in the anaphase nuclei shown in (F).

A major regulator of Cdk1 activity is Cyclin B and degradation of CycB by APC*^Fzy^* is required for exit from mitosis [30]. We next analyzed if degradation of CycB is impaired in *xpd^eE^* embryos. Figure 7 shows an *xpd^eE^* mutant embryo, in which nuclear division synchrony is lost. Nuclei in mitotic phase ranging from prophase to anaphase can be observed in this embryo. We found that the distribution of CycB corresponded well to the division phase of the individual nucleus (Figure 7B–7F). CycB mainly localizes to the spindle pole region of prophase nuclei (arrowheads in Figure 7D). During early metaphase, it is highly concentrated on the entire spindle region (“*” in Figure 7D–7E). The CycB signal on spindle regions then starts to diminish during mid-metaphase (arrowheads in Figure 7E) and then almost drops to cytoplasmic CycB levels when the nuclei are in late metaphase (arrows in Figure 7E). Anaphase nuclei show no regional concentration of CycB (Figure 7F). This correlation between mitotic phase and localization of CycB is similar to that of wild type embryos, although the distribution changes of CycB are synchronized in wild type embryos (data not shown; [31]). It thus seems that CycB can still be degraded during metaphase to anaphase transition. However, in some regions, degradation of CycB is delayed and nuclei in these regions stay longer in mitosis.

If the observed delays are mediated by excessive Cdk1/CycB activity, we may be able to rescue this phenotype by reducing CycB levels in the mutant embryos. To test this, we produced *xpd^eE^* embryos from mothers containing only one wild type copy of *cycB (+/Df* and *+/cycB^2^)* and analyzed the frequency of strong nuclear division defects in the different genetic backgrounds. For this purpose we used two unrelated *cycB* mutant chromosomes, a *Df* chromosome that lacks the region around the *cycB* gene and a chromosome with a point mutation in *cycB*. As strong defects we scored embryos in which a large part of the embryo was affected in cycle 12 or 13 and not only individual nuclei. Embryos of all populations had a similar mitotic index of around 40%. The reduction of maternal *cycB* dose partially rescues the strong nuclear division defects seen in *xpd^eE^* embryos. 5.1% of *xpd^eE^* embryos show such defects (n = 217) and these are fully rescued by a single transgenic copy of genomic *xpd* (n = 108). On the other hand, *xpd^eE^; cycB^2^*/+ embryos show only 2,2% (n = 90) and *xpd^eE^* embryos with only one copy of *cycB* 1,4% of strong nuclear defects (*xpd^eE^; Df*/+; n = 140). While we observe a clear suppression of this phenotype, the metasynchronous division pattern does not get restored in these embryos and, instead, we observe either waves or more irregular division patterns (data not shown).

## Discussion

### NER independent function of Xpd and its role in subcellular localization of CAK

Here, we describe a function for Xpd in the regulation of chromosome segregation in early *Drosophila* embryos that do not depend on transcription for their normal development. A mitotic phenotype caused by the absence of Xpd may also result from defective NER. In this work we have addressed this possibility in different ways, but could not find any evidence for such a defect. In contrary, centrosomes, DNA damage checkpoint and spindle checkpoint appear normal and chromosomes reach normal metaphase configuration, suggesting that the nuclei that show mitotic defects had previously fully replicated their DNA and were not damaged. In addition, high dose UV irradiation (inducing the type of DNA damage that is repaired through NER) of 0–1 hour *xpd^eE^* embryos did not increase the mutant phenotype observed in *xpd^eE^* embryos, even though most of the wild type control embryos treated the same way failed to hatch, which indicates that the UV dose used was efficient (data not shown). Furthermore, the *xpd* mutation *R112H* that displays a compromised NER activity in humans is also sensitive to UV treatment in flies, but embryos from *xpd^P^* mothers rescued with *xpd ^R112H^* do not show increased mitotic defects in early embryonic development with or without UV treatment (Li and Suter, unpublished). It thus appears that the observed phenotypes reflect the direct cell cycle function of Xpd mediated through its physical interaction with CAK. This is also consistent with earlier work that showed that *xpd* negatively regulates the cell cycle function of Cdk7 [10]. This over-expression study showed that high Xpd expression at cycle 13 causes Cdk7 to concentrate in the cytoplasm, away from the chromosomes, and results in skipping of mitosis 13, presumably due to lower cell cycle activity of CAK in the embryo [10]. This experiment already suggested a role of Xpd in localizing Cdk7, and the existence of a quaternary complex with Xpd and CAK (Cdk7/CycH/Mat1) in early Drosophila embryos suggests a direct molecular basis for this process [12],[26]. Now we show that lack of *xpd* prevents the dynamic localization of Cdk7 to unfold properly in *xpd^eE^* embryos (Figure 6). Therefore, Xpd functions to localize Cdk7/CAK to proper cellular compartments and also away from specific compartments. Its proximity to potential substrates may then aid CAK in phosphorylating specific local targets and its removal from specific targets seems to contribute to preventing target phosphorylation as discussed in the next section.

### Regulation of mitotic Cdk activity by Xpd–CAK interaction

Xpd-deprived young embryos show retarded anaphase progression together with reduced polar loss of PH3 signal. This phenotype indicates that Cdk1 activity remains high longer than it should, thereby delaying mitotic exit. As shown in Figure 6D this hyperactivity of Cdk1 correlates with excessive colocalization of Cdk7 signal on the same chromosomes. Because Xpd containing wild type embryos of the same stage show reduced Cdk7 signal in the chromosomal region, this ectopically localized Cdk7 is at present the best candidate for what mediates the excessive Cdk1 activity caused by lack of Xpd. How could then faulty localization of Cdk7 affect local inactivation of Cdk1 activity? Cdk7 is the in vivo Cdk activating kinase (CAK) in metazoa that phosphorylates a Threonine residue in the T-loop of Cdk1, thereby activating this kinase [2]. In human cells there is good evidence that Cdk1 gets dephosphorylated in the T-loop in every cycle and needs to be rephosphorylated by Cdk7 in order to allow entry into mitosis [1]. Experiments with fission yeast indicate that the dephosphorylation of the T-loop Thr of Cdk1 is actually required for mitotic exit [32]. Over-expression of Cdk1 with Thr161 substituted by a glutamate, which mimics T-loop phosphorylation, caused cell division defects in anaphase. A clear reduction in T-loop phosphorylation of Cdk1 is also seen in early interphase Drosophila embryos during the later nuclear division cycles, at the time when manipulating Cdk7 (CAK) activity interferes with normal mitosis [10],[19]. Here we now report a good correlation between local depletion of Cdk7 from chromosomes exiting mitosis and reduction of mitotitc kinase activity on these chromosomes (Figure 6D). The combined results therefore suggest that T-loop dephosphorylation of Cdk1 is also important in Drosophila embryos for mitotic exit, that dephosphorylation may take place locally on the chromosomes and that this is supported by local depletion of CAK.

### Xpd as a chromosomal bodyguard

Many *xpd^eE^* nuclear division defects show similarities to described cell cycle mutants such as *wee1*, *cycB*, *cycB*-stabilized, *grapes*, *bubR1*, *INCENP*/*aurora B*, *vHL*, and *kinesin-5* [29], [33]–[40]. Many of these genes also function in controlling microtubule spindles. The work presented here now adds *xpd* to the group of genes that control aspects of spindle dynamics. However, while certain aspects of the *xpd^eE^* phenotypes overlap with phenotypes of the other mutants, others do not. For instance we do not observe differences in spindle length between rescued and *xpd^eE^* mutants in metaphases 10–13 and also no obvious reduction in astral microtubules as had been described for changes in *cycB* activity (data not shown; [37]). This indicates that *xpd* does not simply act as a component of a linear control pathway. A model in which the Xpd polypeptide performs its cell cycle function by localizing the protein kinase Cdk7/CAK to and away from specific subcellular compartments and target proteins can better explain the observed combination of various mitotic phenotypes in *xpd^eE^* embryos. Interestingly, promiscuous spindles as described for *xpd^eE^* mutants (Figure 5) were also seen in *wee1* mutants and in kinesin-5 mutants where the Wee1 target tyrosines were replaced and this suggested a control pathway [33],[34]. Because the mitotic kinases Cdk1 and Polo repress Wee1 kinase [41], it appears that lack of Xpd could cause this phenotype by causing locally elevated mitotic kinase activity that prevents Wee1 from acting on kinesin-5.

The misappropriation of chromosomes by neighboring spindles in *xpd^eE^* embryos points to novel functions of *xpd* that appear relevant for the understanding of its physiological function and the defects seen in human *XPD* patients. In *xpd^eE^* embryos microtubules from one mitotic spindle frequently reach into the territory of a neighboring spindle, while in the wild type they appear to avoid neighboring territories (Video S1 and Video S2). In the case of the syncytium studied here this can lead to the attachment of a chromosome from the wrong nucleus and to multipolar spindles and ensuing problems. In a mononuclear diploid cell such a spindle problem may also lead to spindle orientation defects during mitosis, a function that is crucial for many division processes, including the development of the nervous system. In addition, in an interphase cell proper guidance and targeting of microtubule minus ends is crucial for neuronal development and functioning. The phenotypes described here may therefore also provide the basis for a better understanding of the human neurological deficits associated with mutant alleles of *xpd*.

## Materials and Methods

### Analysis of the *l(2)SH2137 xpd* line and in vivo functionality of *xpd* constructs

*Df(2R)K11* has region 57C3–57D8 deleted. The *xpd* P-element insertion line *l(2)SH2137* [42] was characterized by PCR amplification of its genomic DNA using a primer that anneals to the fourth exon of *xpd* and one that anneals to the 5′-end of *P*[lacW]. Resulting PCR products were then sequenced. Wild-type, V5- and GFP- or YFP-tagged *xpd* transgenes inserted on the X or 3^rd^ chromosome were tested for their ability to complement *l(2)SH2137* by crossing flies trans-heterozygous for the transgene and *Df(2R)K11* to *w*; *xpd^P^*/CyO flies and scoring for progeny of the second chromosome genotype *xpd^P^*/*Df(2R)K11*, that were obtained only in the presence of a rescuing transgene.

### DNA cloning and constructs

From the Drosophila genomic clone BACR16B07 [43] an 8.5 kb DNA fragment containing the *xpd* genomic DNA and two adjacent predicted genes was cut out with EcoRI and NheI. The two adjacent genes were removed with an RsrII digestion. The resulting 5.9 kb fragment was cloned into the pCaSpeR transformation vector. To construct *xpd-V5*, *xpd-GFP* and *xpd-YFP*, the stop codon of the *xpd* gene was mutated to introduce an XbaI site. *V5* tag, from the pMT/V5-His vector (Invitrogen, San Diego, CA), *GFP*, from pTracer-CMV/Bsd (Invitrogen), and *YFP*, from peYFP vector (Clontech, Mountain View, CA) were then cloned into the XbaI site. For the *xpd* cDNA construct, the same strategy as above was applied to fuse the V5 tag to the C-terminal end of the *xpd* ORF. *xpd-V5* was then cloned into the pUAST vector.

### Fly stocks and special chromosomes

*tub-Gal4* line (*y w; tub-Gal4/TM3 Sb*) is from the Bloomington Stock Center (No. 5138) and *G147* from Morin et al. [25]. Both were recombined onto the same chromosome. *xpd* genomic DNA alone, or fused with a *V5* tag, a *GFP* gene or a *YFP* gene were cloned into the pCaSpeR vector. *xpd-V5* cDNA was cloned into pUAST and the genomic and cDNA constructs were transformed into *y w* flies. *xpd* genomic DNA alone was also inserted site-specifically into landing platform 64A (K. Stettler, pers. communication) [44], and the resulting chromosome was recombined with the *G147* chromosome. To produce *xpd^eE^* embryos with only one functional copy of *cycB*, we isolated recombinants between the *cycB^2^* [45] and Df(2R)59AB [46] on the one hand and the *xpd^P^* chromosome.

### Immunostaining and image analysis

Embryo staining with anti-PH3, -Cdk7, -CycB and -γ tubulin was performed essentially as described [10],[31], except that for Cdk7 staining devitellinization was done manually and not with methanol. For anti-Tubulin staining, methanol fixation was performed to preserve microtubule structures. The following antibodies were used for embryo immunostaining at indicated conditions: monoclonal anti-Cdk7 at 1/5 (a combination of 20H5 and 10E10 2; [2]), polyclonal anti-CNN at 1/600 [47], polyclonal anti-PH3 at 1/100 (Cell Signaling Technology, Beverly, MA), anti-V5 (1/100; Invitrogen, San Diego, CA), polyclonal anti-CycB (1/30; [31]), and monoclonal anti-α and β tubulin at 1/100 (AA4.3 and AA12.1 from the developmental studies hybridoma bank, the University of Iowa), monoclonal anti-γ tubulin at 1/500 (clone GTU-88, Sigma). The embryos and ovaries were observed under a Leica DM6000 fluorescence microscope or a Leica TCS-SP2 confocal laser scanning microscope. Signal intensities in confocal images were measured for each channel along a 14 µm straight line parallel to the spindle axis of individual nuclei, using the quantification function of the Leica Application Suite 2.0.2 (Leica Microsystems, Mannheim, Germany). The resulting data was exported into Microsoft Excel to average the measurements of 5 nuclei per image. A moving average trend-line with a period of 20 was then inserted for each channel. However, one obtains qualitatively indistinguishable results by directly printing out the graphs from the Leica Application Suite and averaging the graphs manually (data not shown).

### Live imaging

Live imaging was performed essentially as described by Cavey and Lecuit [48]. Briefly, *xpd^eE^* embryos from *w; xpd^P^; tub-gal4 G147/UAST-xpd-V5* mothers, *w; xpd^P^/CyO; tub-gal4 G147/UAST-xpd-V5* control mothers or *w; xpd^P^; xpd^+^*(/*TM3*) rescue mothers were collected on apple juice plates, dechorionated for 1–2 min in 2.5% bleach, washed in tap water (being careful not to cause hypoxia by prolonged submersion in water), glued onto cover slips prepared with heptane/tape glue mix, and covered with Voltalef oil 3S (VWR, Fontenay, France). Images were acquired using a Leica TCS-SP2 confocal system with an inverted microscope stand, and a 488 nm laser to excite GFP. Each image was 1024×1024 pixels, averaged 4times (line average), with an interval of 30 sec between consecutive images.

### mRNA synthesis and injection into embryos

*xpd* ORF was PCR amplified from an *xpd* cDNA clone [49] using the primer pair 5′-CCGAGCTCTAAATGAAAGTACTCCTTAA-3′ (underlined SacI site) and 5′- GAAGATCTTCACAGCTCCTGCACCTCGC-3′ (underlined BglII site), and *myc-eCFP* ORF was amplified from c-myc-CFP-pBS (gift from P. Vazquez) using the primer pair 5′-CATGCCATGGAGCAAAAGCTCATTTCTG-3′ (underlined NcoI site) and 5′-GAAGATCTCACTTGTACAGCTCGTCCATGCCGAG-3′ (underlined BglII site). The amplification products were cloned with SacI/BglII and NcoI/BglII, respectively, into a pBluescript vector with a poly-A sequence (73 As, gift from P. Vazquez). One clone for each construct was selected and sequenced. The plasmids were linearized downstream of the polyA sequence with XhoI, and gel-purified using QIAquick Gel extraction columns from Qiagen (Hilden, Germany). 1 µg of these DNA templates were used for in vitro transcription with an Ampliscribe T3 High Yield Transcription Kit (Epicentre Biotechnologies, Madison, WI), using an RNA Cap Structure analog from New England Biolabs (Ipswich, MA). This was followed by a DNaseI (Epicentre Biotech.) digest. RNAs were purified with RNeasy columns (Qiagen) and eluted in RNase-free water (Epicentre Biotech.). RNAs were concentrated to approximately 400 ng/µl by drying, and verified on agarose gels. Injection was done ventrally into 20–45 min old *xpd^eE^* embryos with an Eppendorf Femtojet microinjector (Hamburg, Germany), using glass needles produced from Drummond capillaries (Broomall, PA; needle puller: Sutter instruments; Novato, CA).

### Western blotting and CAK assay

For Western blotting, the following antibodies were used at indicated concentration at 4°C for over night or at room temperature for 1 hr. Anti-Grp (1/500), anti-HA (1/2000), monoclonal anti-CycB (1/500; DSHB), anti-Cdc2/anti-PSTAIR (1/1500), monoclonal anti-Cdk7 (1/10), polyclonal anti-Xpd (affinity purified, 1/1000; [10]), anti-V5 (1/2000; Invitrogen, San Diego, CA). For Western blots, transfer was onto nitrocellulose membranes and signal detection was done with the ECL plus system (GE Healthcare, UK). Quantification of signals was performed with the ImageJ software [50]. CAK assays were carried out as described previously [2].

## Supporting Information

Figure S1 Lack of Xpd does not cause centrosome inactivation. Proper γ-tubulin localization to the centrosomes of a cycle 12 *xpd^eE^* embryo with delayed histone H3 de-phosphorylation in anaphase. Scale bar represents 10 µm.
(1.85 MB PDF)

Table S1 (1) Only the genotype of the 2^nd^ chromosome is indicated. “Df” stands for *Df(2R)K11*. The flies are the offspring of the cross *w; xpd^P^/CyO* females crossed with *w P[w^+^ xpd^+^]/*Y; *Df(2R)K11/b Tft* males. “*w*”: X chromosome carrying a recessive white eye color mutation. “Y”: Y chromosome. “*CyO*”: Balancer for the second chromosome preventing recombination and carrying a dominant visible marker mutation. “*P[w* ^+^ *xpd^+^]*”: *P* transposable element carrying a minigene conferring reddish eye color for visual selection in a “*w* background”, and a transgenic copy of *xpd^+^* under control of its predicted endogenous promoter. The transgenic *xpd^+^* is a wild type allele without tag and with a V5-tag, respectively. “*b Tft*”: chromosome with visible marker mutations. (2) For the females, the expected frequencies of the different genotypes are shown for the case that the *xpd^+^* transgenes do not rescue hemizygous *xpd^P^* (*) and the case that they fully rescue it (**). The hemizygous *xpd^P^* genotype (*xpd^P/−^*) is colored in green. (3) The males serve as negative controls as they inherit from their fathers the Y chromosome and not the X chromosome carrying the rescue construct. n = 2057 for the rescue with *xpd^+^* and n = 906 for the rescue with V5-tagged *xpd^+^*.
(0.30 MB DOC)

Video S1 Spindle dynamics of wild-type embryos. Movie of G147::GFP expression in wild-type embryos.
(10.18 MB MPG)

Video S2 Spindle dynamics and lack of division coordination in an *xpd^eE^* embryo. Movie of G147::GFP expression in *xpd^eE^* embryos.
(9.95 MB MPG)
