# Supplementary material for: Drosophila Xpd Regulates Cdk7 Localization, Mitotic Kinase Activity, Spindle Dynamics, and Chromosome Segregation
Source: PLoS Genet. 2010 Mar 12;6(3):e1000876. doi: 10.1371/journal.pgen.1000876 (PMC2837399; doi:10.1371/journal.pgen.1000876)
Supplement: Figure S1 — Lack of Xpd does not cause centrosome inactivation. Proper γ-tubulin localization to the centrosomes of a cycle 12 xpdeE embryo with delayed histone H3 de-phosphorylation in anaphase. Scale bar represents 10 µm. (1.85 MB PDF) [file pgen.1000876.s001.pdf]

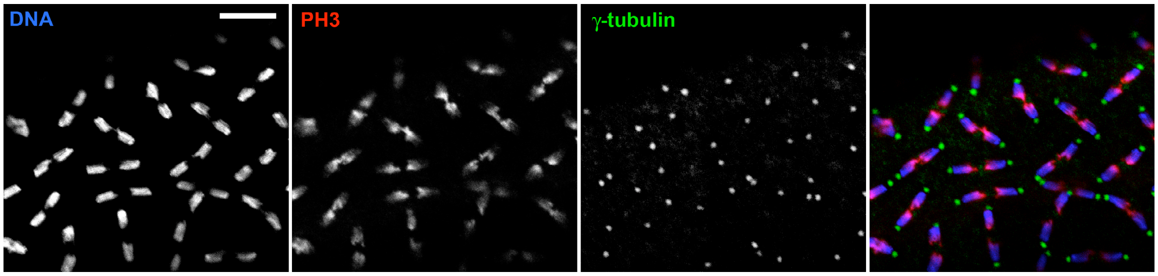

Figure S1: Lack of Xpd does not cause centrosome inactivation. Proper γ-tubulin localization to the centrosomes of a cycle 12 xpd^{eE} embryo with delayed histone H3 de-phosphorylation in anaphase. Scale bar represents 10 μm.
