# Supplementary material for: Drosophila Xpd Regulates Cdk7 Localization, Mitotic Kinase Activity, Spindle Dynamics, and Chromosome Segregation
Source: PLoS Genet. 2010 Mar 12;6(3):e1000876. doi: 10.1371/journal.pgen.1000876 (PMC2837399; doi:10.1371/journal.pgen.1000876)
Supplement: Table S1 — (1) Only the genotype of the 2nd chromosome is indicated. “Df” stands for Df(2R)K11. The flies are the offspring of the cross w; xpdP/CyO females crossed with w P[w+ xpd+]/Y; Df(2R)K11/b Tft males. “w”: X chromosome carrying a recessive white eye color mutation. “Y”: Y chromosome. “CyO”: Balancer for the second chromosome preventing recombination and carrying a dominant visible marker mutation. “P[w + xpd+]”: P transposable element carrying a minigene conferring reddish eye color for visual selection in a “w background”, and a transgenic copy of xpd+ under control of its predicted endogenous promoter. The transgenic xpd+ is a wild type allele without tag and with a V5-tag, respectively. “b Tft”: chromosome with visible marker mutations. (2) For the females, the expected frequencies of the different genotypes are shown for the case that the xpd+ transgenes do not rescue hemizygous xpdP (*) and the case that they fully rescue it (**). The hemizygous xpdP genotype (xpdP/−) is colored in green. (3) The males serve as negative controls as they inherit from their fathers the Y chromosome and not the X chromosome carrying the rescue construct. n = 2057 for the rescue with xpd+ and n = 906 for the rescue with V5-tagged xpd+. (0.30 MB DOC) [file pgen.1000876.s002.doc]

**Table S1:**

**Rescue of lethality of *xpd^P/-^* mutants by transgenic V5-tagged or untagged *xpd^+^***

**
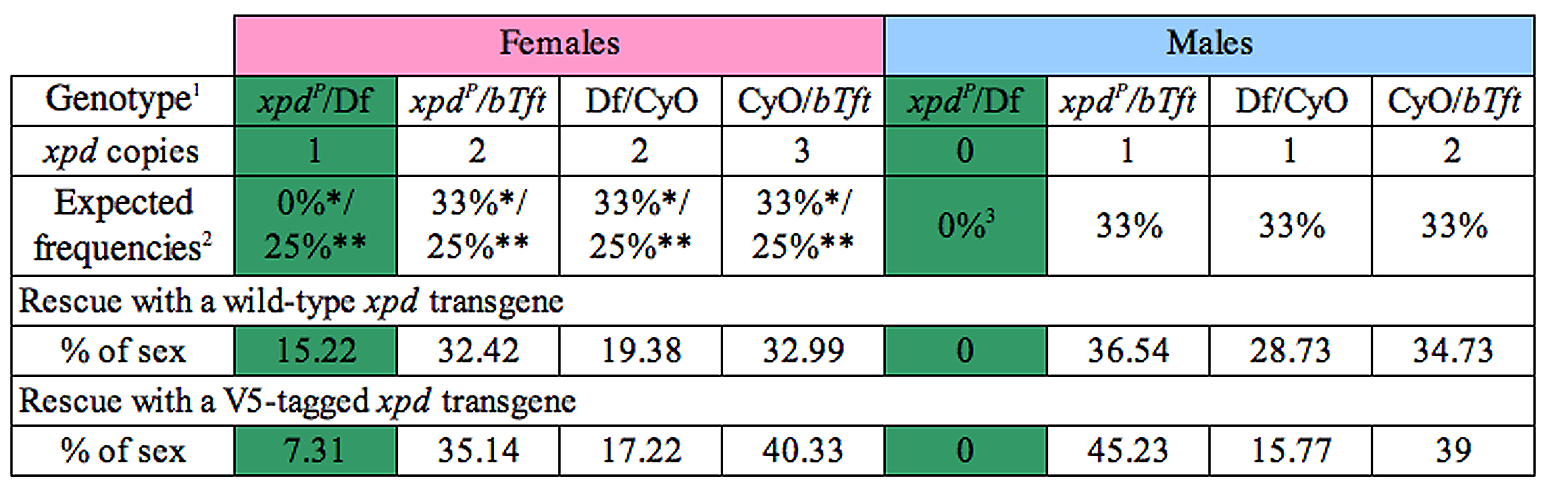
**

(^1^) Only the genotype of the 2^nd^ chromosome is indicated. „Df“ stands for *Df(2R)K11*. The flies are the offspring of the cross *w; xpd^P^ / CyO* females crossed with *w P[w^+^ xpd^+^] /* Y*; Df(2R)K11 / b Tft* males. „*w*“: X chromosome carrying a recessive white eye color mutation. “Y”: Y chromosome. „*CyO*“: Balancer for the second chromosome preventing recombination and carrying a dominant visible marker mutation. „*P[w*^+^ *xpd^+^]*“: *P* transposable element carrying a minigene conferring reddish eye color for visual selection in a “*w* background”, and a transgenic copy of *xpd^+^* under control of its predicted endogenous promoter. The transgenic *xpd^+^* is a wild type allele without tag and with a V5-tag, respectively. “*b Tft*”: chromosome with visible marker mutations.

(^2^) For the females, the expected frequencies of the different genotypes are shown for the case that the *xpd^+^* transgenes do not rescue hemizygous *xpd^P^* (*) and the case that they fully rescue it (**). The hemizygous *xpd^P^* genotype (*xpd^P/-^*) is colored in green.

(^3^) The males serve as negative controls as they inherit from their fathers the Y chromosome and not the X chromosome carrying the rescue construct.

n=2057 for the rescue with *xpd^+^* and n=906 for the rescue with V5-tagged *xpd^+^*.
